# Supplementary material for: mRNA Therapeutic Vaccine for Hepatitis B Demonstrates Immunogenicity and Efficacy in the AAV-HBV Mouse Model
Source: Vaccines (Basel). 2024 Feb 25;12(3):237. doi: 10.3390/vaccines12030237 (PMC10976109; doi:10.3390/vaccines12030237)

# Supplementary Figure

**S1: Gating strategy for intracellular multicolor flow panel for determination of HBV-specific polyfunctional T-cells**

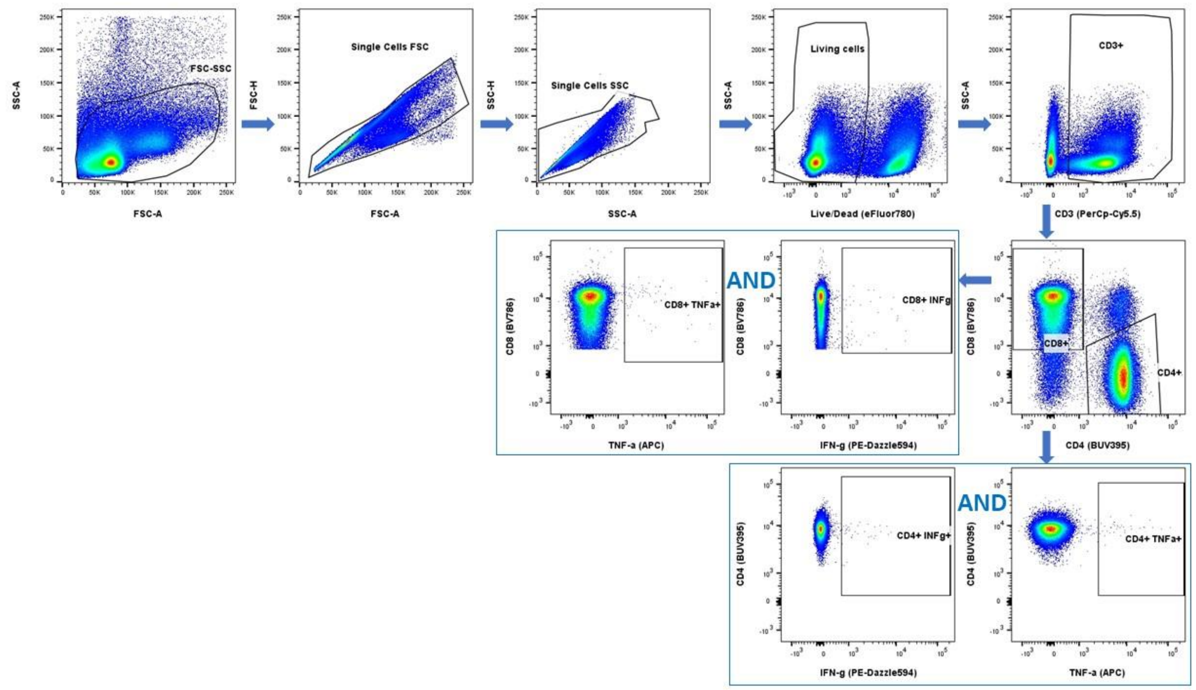

**S2: Evaluation of induced immune responses in naive C57BL/6 mice after vaccination with co-formulated (MC3) mRNA vaccine encoding for three HBV antigens compared to for formulated mRNA encoding for one HBV antigen.**

Induced immune responses after prime/boost, as measured by IFN-γ ELISpot (vertical axis) for co-formulated (triangle) versus single antigen vaccine (black circles) at 5 μg per mRNA after stimulation with different peptide pools (horizontal axis) (n=6).

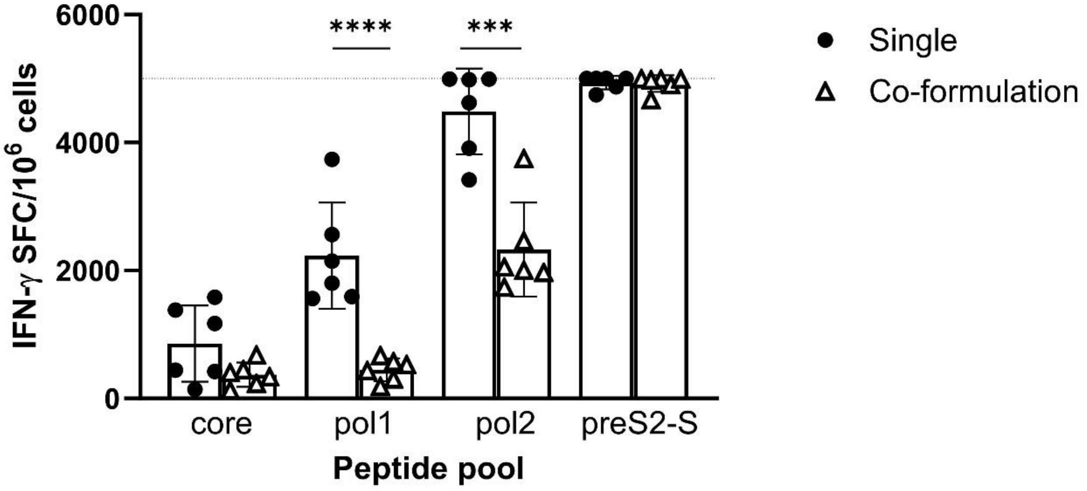


**S3: Evaluation of induced anti-HBe in naive C57BL/6 mice after vaccination with coformulated mRNA vaccine in dose response.**

Induced concentrations of anti-HBe (vertical axis), measured by CLIA, at day 14 and 28 (horizontal axis) after different doses (15 μg white, 6 μg light grey and 3 μg dark grey) of coformulation (n=6).

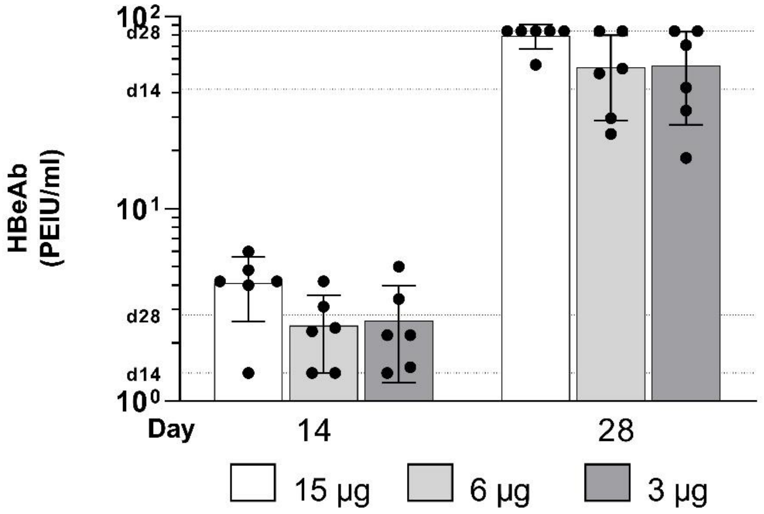


**S4: Evaluation of induced anti-HBe in naive BALB/c mice after vaccination with co-encapsulated mRNA vaccine.**

Induced concentrations of anti-HBe (vertical axis), measured by CLIA, at day -1, 14, 21 and 28 (horizontal axis) for vaccinated mice (black) versus saline control (grey) (n=6).

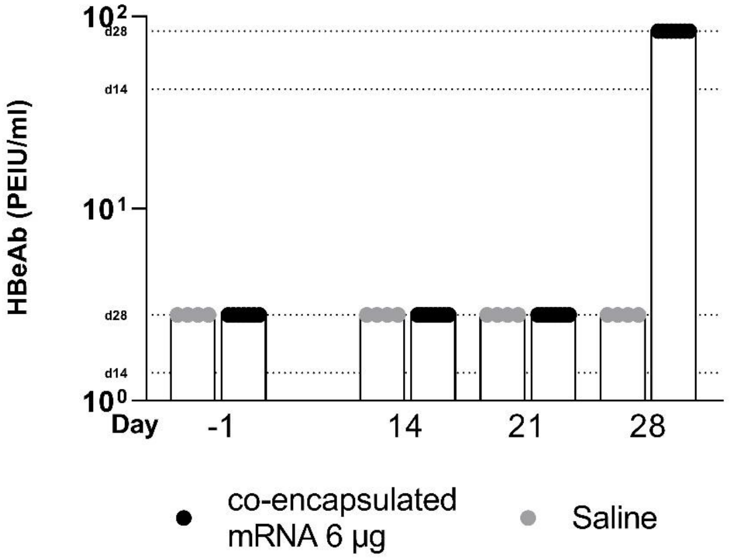

**S5: Immunohistochemistry staining for HBsAg in AAV-HBV transduced mice in saline control group.**

HBsAg positive areas are indicated in brown against the blue colored cells for each mouse of the saline treated group.

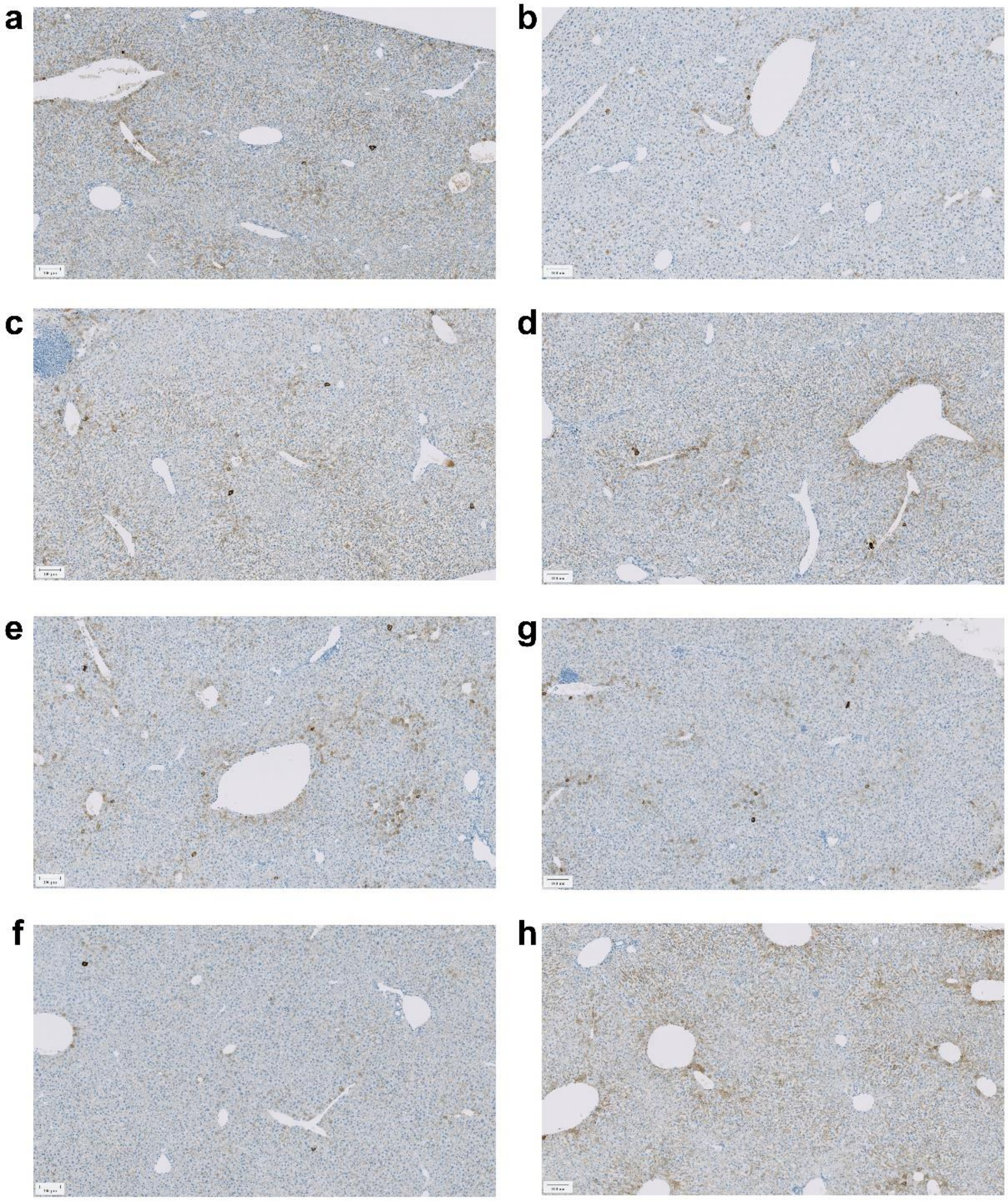

**S6: Immunohistochemistry staining for HBsAg in AAV-HBV transduced mice in mRNA vaccinated group.** HBsAg positive areas are indicated in brown against the blue colored cells for each mouse of the mRNA vaccinated group with corresponding symbols from figure 6d-e.

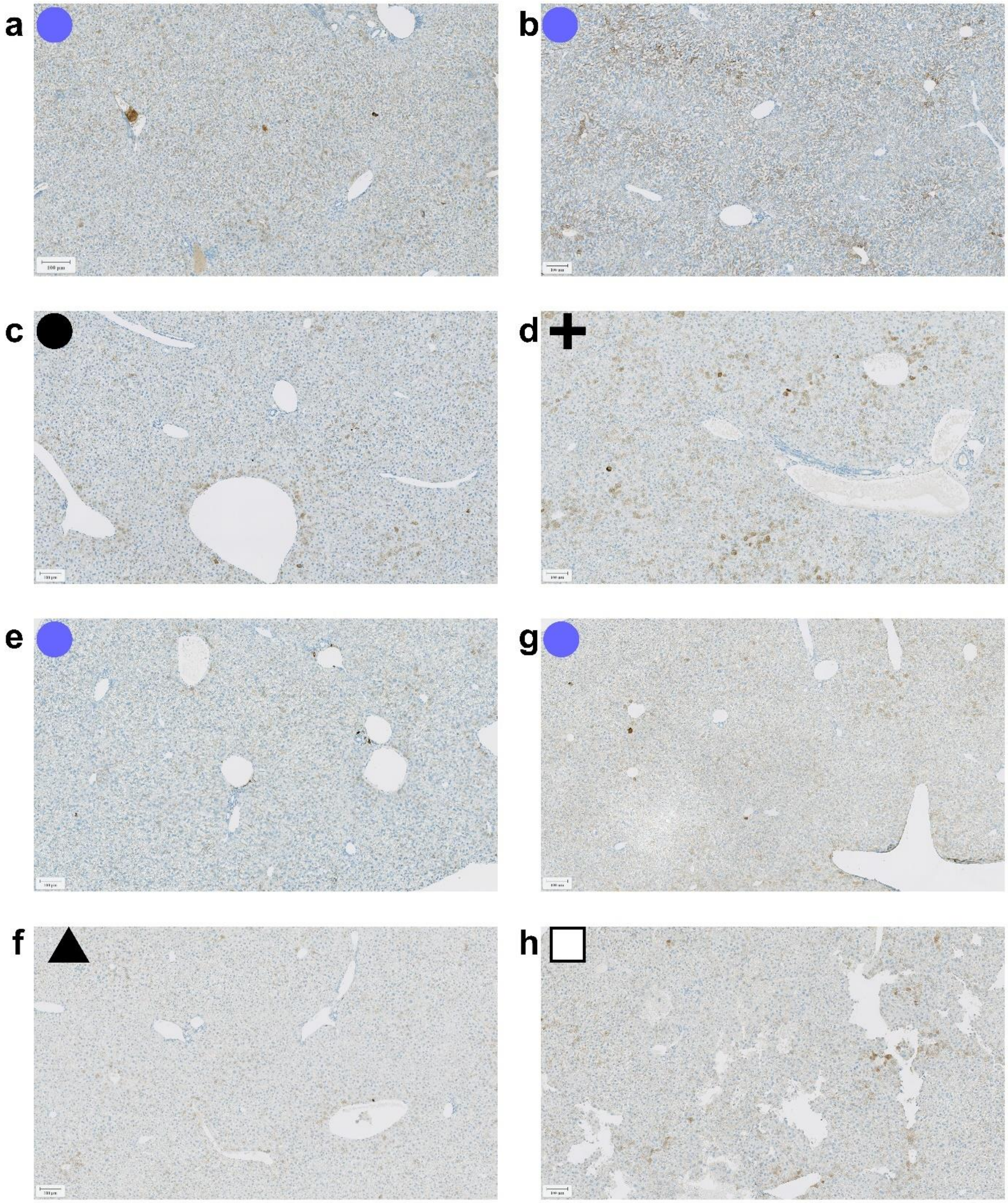

**S7: Immunohistochemistry staining for HBsAg in AAV-HBV transduced mice in saline control group.**

HBsAg positive areas are indicated in brown against the blue colored cells for each mouse of the saline treated group.

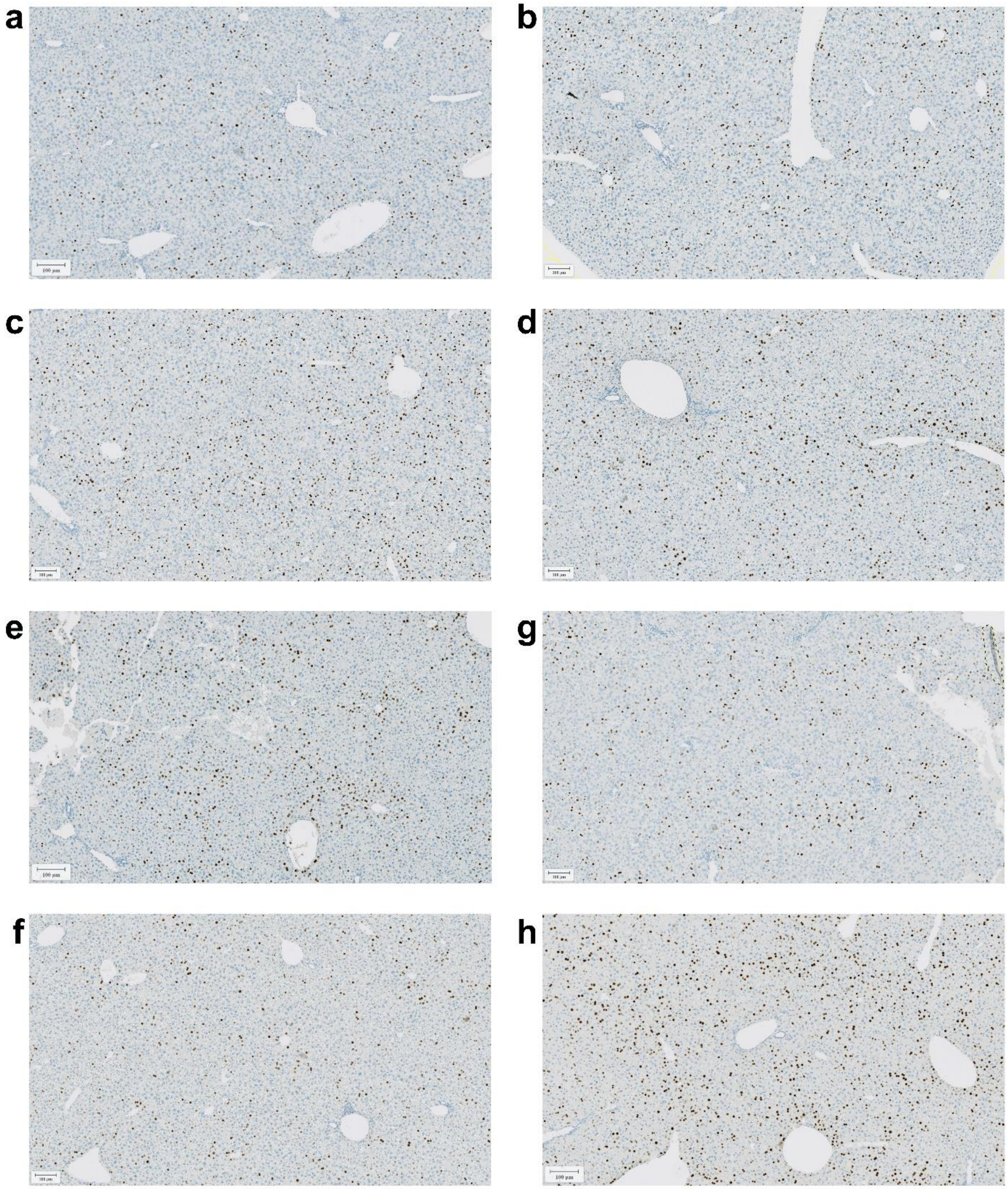

**S8: Immunohistochemistry staining for HBcAg in AAV-HBV transduced mice in mRNA vaccinated group.**

HBcAg positive cells are indicated in brown against the blue colored cells for each mouse of the mRNA vaccinated group with corresponding symbols from figure 6d-e added.

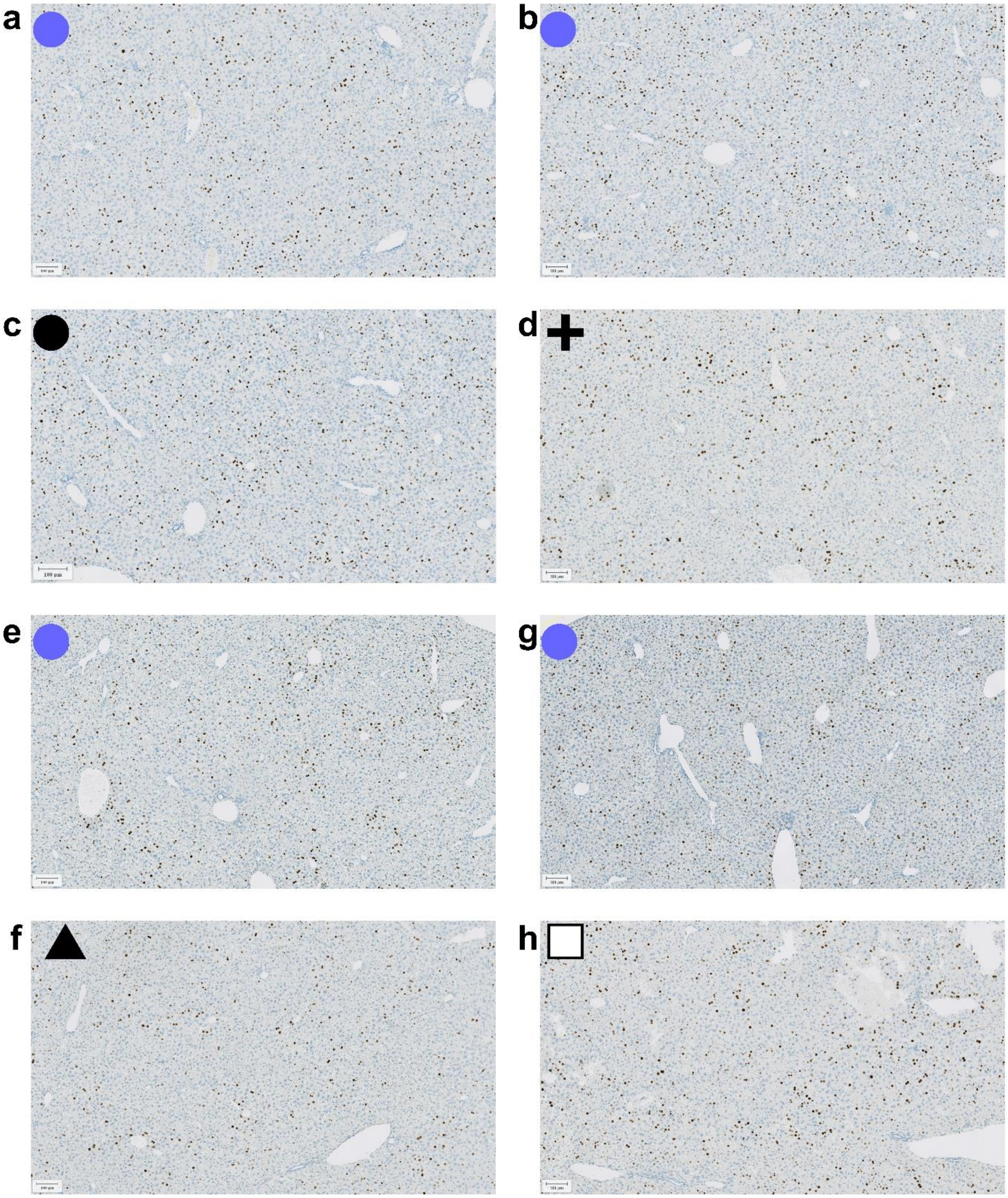

Supplement: Supplementary file 1 [file vaccines-12-00237-s001.zip › mRNA TxVx_DorienDePooter_Suppl.Fig_20240207.pdf]
