# Supplementary figures and images for: mRNA Therapeutic Vaccine for Hepatitis B Demonstrates Immunogenicity and Efficacy in the AAV-HBV Mouse Model
Source: Vaccines (Basel). 2024 Feb 25;12(3):237. doi: 10.3390/vaccines12030237 (PMC10976109; doi:10.3390/vaccines12030237)

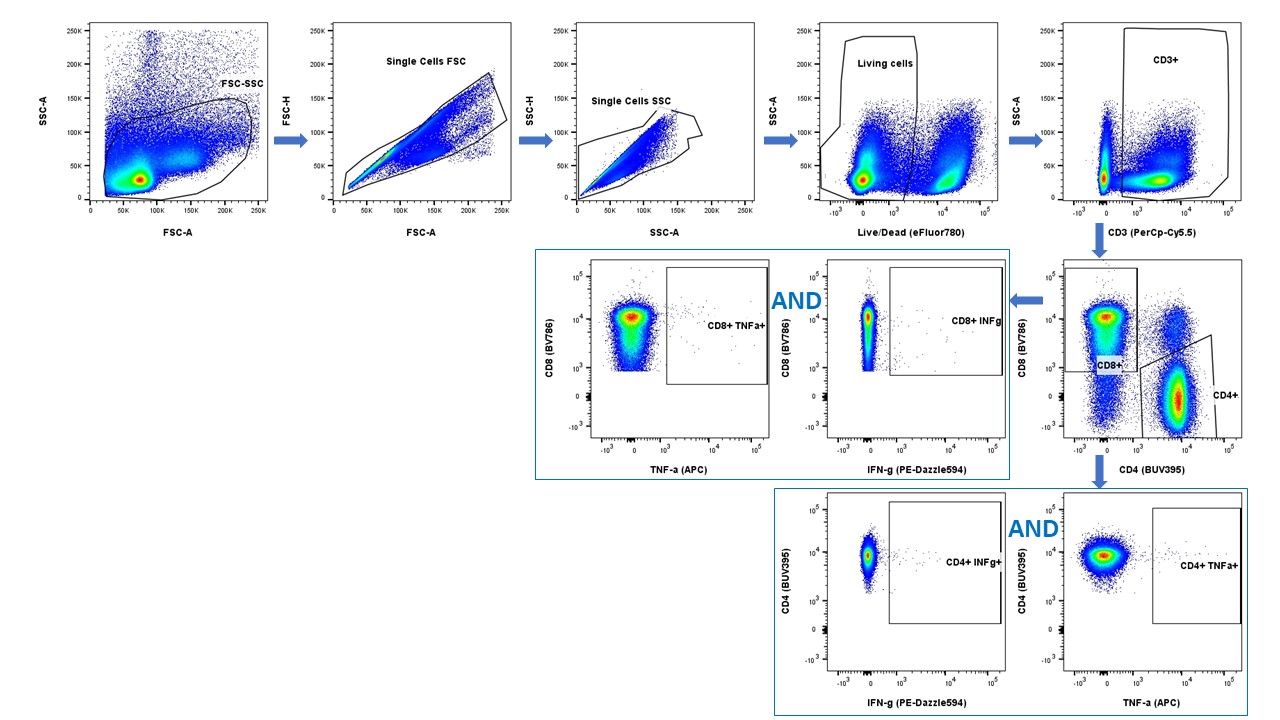

Supplement: Supplementary file 1 [file vaccines-12-00237-s001.zip › Figure S1.JPG]

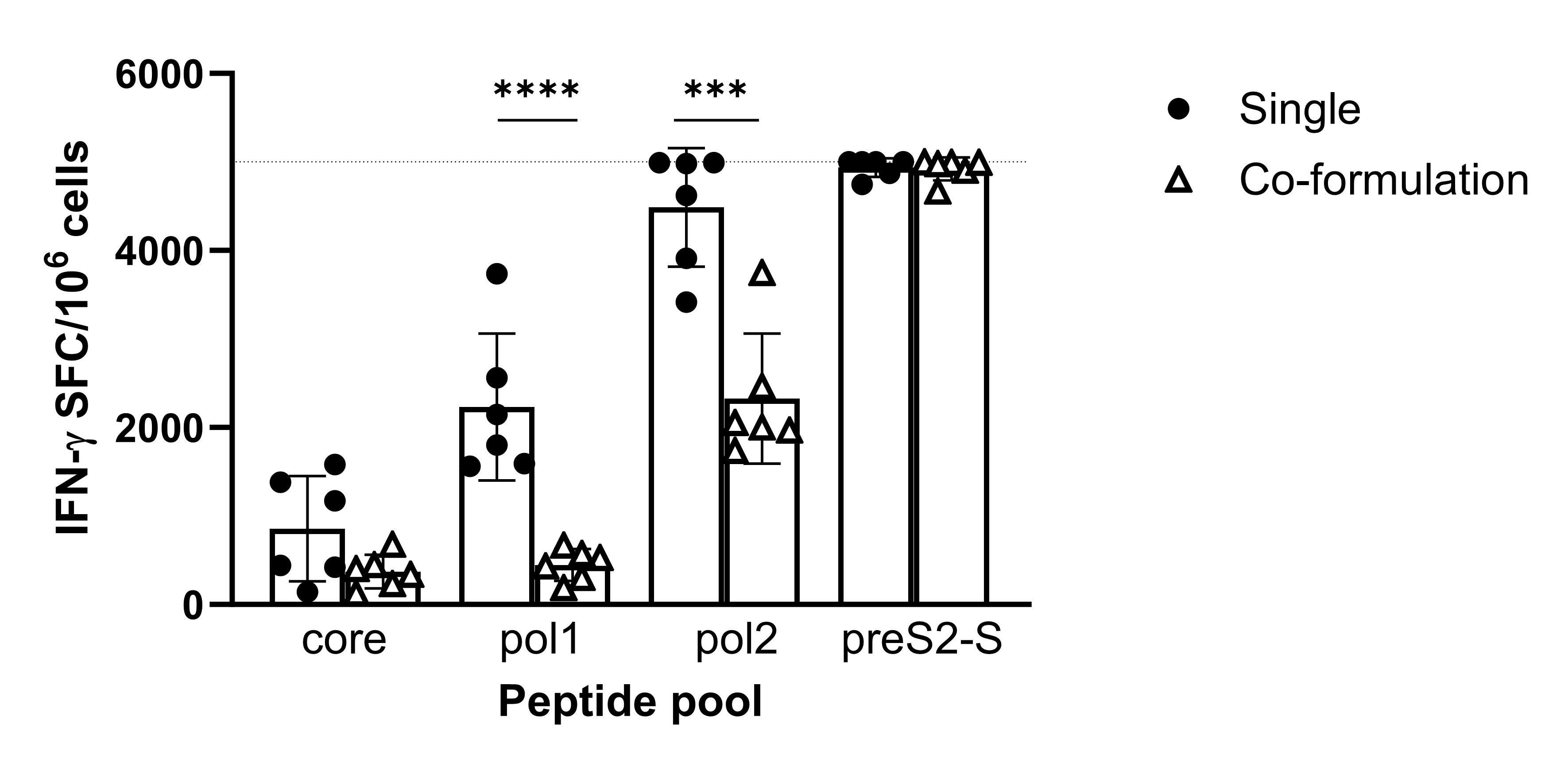

Supplement: Supplementary file 1 [file vaccines-12-00237-s001.zip › Figure S2.jpg]

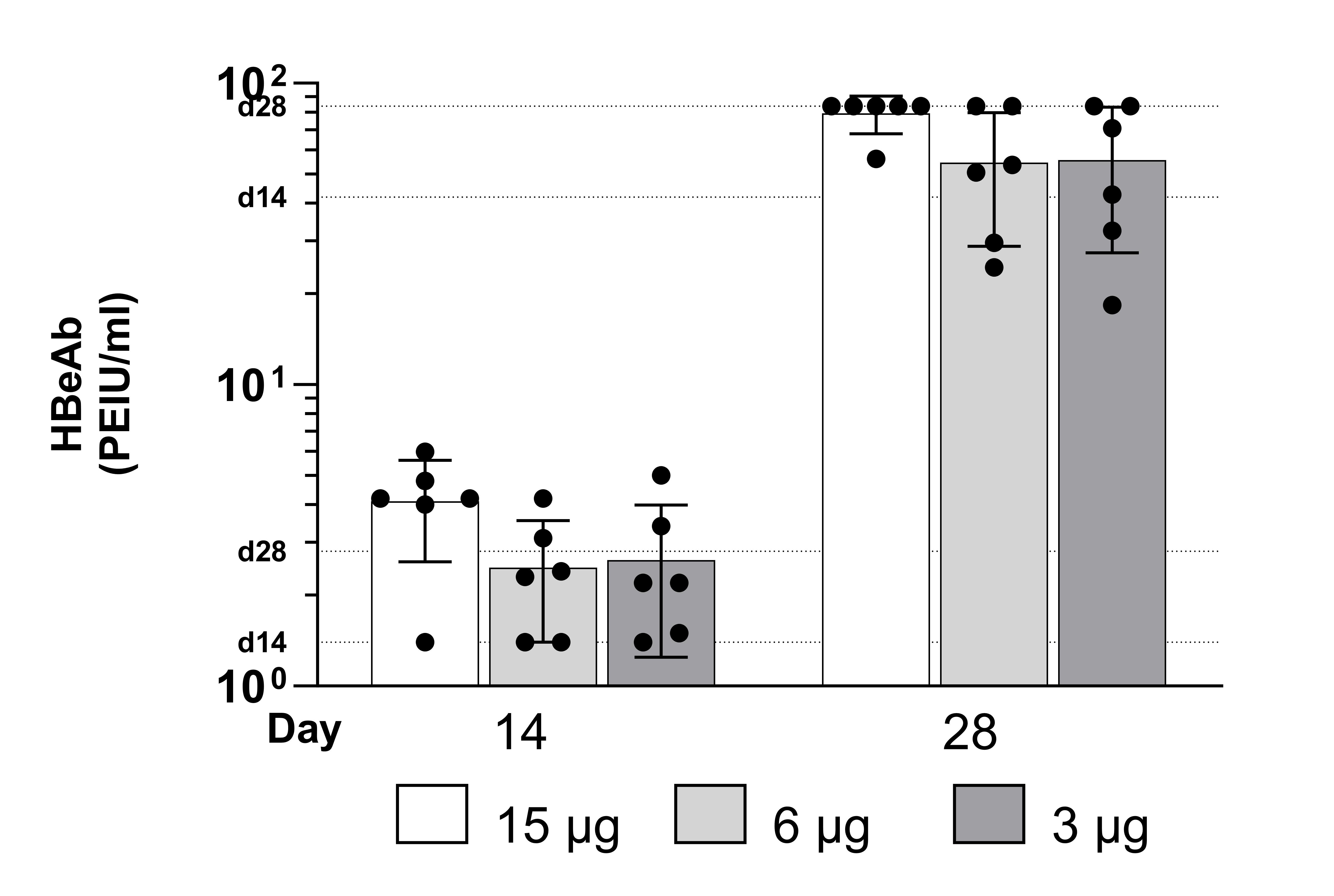

Supplement: Supplementary file 1 [file vaccines-12-00237-s001.zip › Figure S3.jpg]

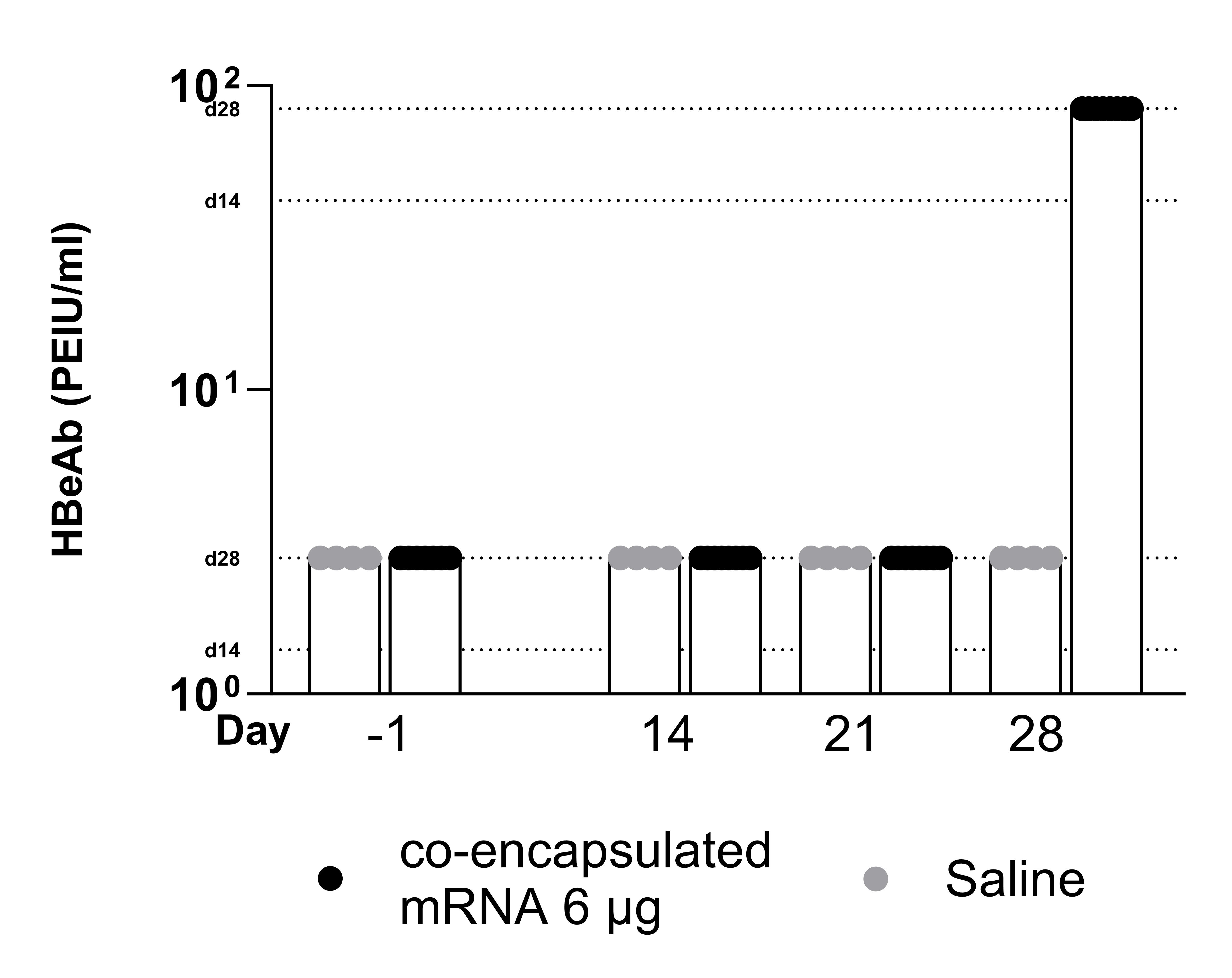

Supplement: Supplementary file 1 [file vaccines-12-00237-s001.zip › Figure S4.jpg]
